# Construction of the Coding Sequence of the Transcription Variant 2 of the Human Renalase Gene and Its Expression in the Prokaryotic System

**DOI:** 10.3390/ijms140612764

**Published:** 2013-06-19

**Authors:** Valerii I. Fedchenko, Alexei A. Kaloshin, Lyudmila M. Mezhevikina, Olga A. Buneeva, Alexei E. Medvedev

**Affiliations:** 1 Orekhovich Institute of Biomedical Chemistry, Russian Academy of Medical Sciences, 10 Pogodinskaya Street, Moscow 119121, Russia; E-Mails: valfed@ibmc.msk.ru (V.I.F.); Alex-k-1973@yandex.ru (A.A.K.); olbun@yandex.ru; olbun@yandex.ru (O.A.B.); 2 Institute Cell Biophysics, Russian Academy of Sciences, 3 Institutskaya Street, Pushchino, Moscow Region, Moscow 142290, Russia; E-Mail: mezhevikina@rambler.ru

**Keywords:** human renalase gene, transcription variants, PCR-based exon joining, recombinant protein, western blot analysis

## Abstract

Renalase is a recently discovered protein, involved in regulation of blood pressure in humans and animals. Although several splice variants of human renalase mRNA transcripts have been recognized, only one protein product, hRenalase1, has been found so far. In this study, we have used polymerase chain reaction (PCR)-based amplification of individual exons of the renalase gene and their joining for construction of full-length hRenalase2 coding sequence followed by expression of hRenalase2 as a polyHis recombinant protein in *Escherichia coli* cells. To date this is the first report on synthesis and purification of hRenalase2. Applicability of this approach was verified by constructing hRenalase1 coding sequence, its sequencing and expression in *E. coli* cells. hRenalase1 was used for generation of polyclonal antiserum in sheep. Western blot analysis has shown that polyclonal anti-renalase1 antibodies effectively interact with the hRenalase2 protein. The latter suggests that some functions and expression patterns of hRenalase1 documented by antibody-based data may be attributed to the presence of hRenalase2. The realized approach may be also used for construction of coding sequences of various (especially weakly expressible) genes, their transcript variants, *etc*.

## 1. Introduction

Renalase is a recently discovered protein, which is likely involved in regulation of blood pressure in humans and animals [1–4]. Certain evidence also exists that circulating renalase reflects kidney functioning [3]. Although mechanisms responsible for the renalase-mediated normalization of blood pressure remain unknown [1,2] and pilot studies on the involvement of renalase in degradation of circulating catecholamines [2,5] have not been confirmed by other laboratories [1,6,7], there is increasing evidence that renalase is a novel important regulator of blood pressure [1,3].

According to information available in GenBank (http://www.ncbi.nlm.nih.gov/sites/entrez?db=gene&cmd=Retrieve&dopt=full_report&list_uids=55328) [8], the renalase gene is located on chromosome 10 at q23.31 and consists of 309,462 bp (NC_000010.10). Automated computational analysis validated by corresponding mRNA sequencing gives two mRNA variants designated as the transcript variant 1 (NM_001031709.2) and the transcript variant 2 (NM_018363.3), which encode putative protein products designated as renalase isoform 1 precursor (NP_001026879.2) and renalase isoform 2 precursor (NP_060833.1), respectively. These two isoforms are also known as hRenalase1 and hRenalase2, respectively.

The transcript variant 1 (1477 nucleotides (nt)) contains seven exons and encodes a protein (hRenalase1) of 342 residues with calculated molecular mass of 37.85 kDa. This transcript variant was preferentially found in the glomeruli and proximal tubules, and also in cardiomyocytes, liver, and skeletal muscles [5]. More recently, other authors detected hRenalase1 mRNA in peripheral nerves, adrenal and adipose tissue [9].

The transcript variant 2 (2107 nt) contains five exons (2/6) identical to the transcript variant 1 and differs by the last, seventh, exon; it also has a single nucleotide substitution, which determines amino acid substitution Glu37→Asp in the first exon as compared to the amino acid sequence of hRenalase1. This transcript variant 2 encodes a protein (hRenalase2) of 315 residues with calculated molecular mass of 34.95 kDa. In the very first publication on renalase, an mRNA species of about 2.4 kb similar in length to the transcript variant 2 was identified in skeletal muscles [5]. Using real-time polymerase chain reaction (RT-PCR) several renalase-specific transcripts were detected in human adrenal glands, as well as the left ventricle and hypothalamus [9].

However, except for hRenalase1, protein products encoded by corresponding renalase mRNAs have not yet been identified and their biological role remains unknown. It is possible that some results based on ELISA analysis of plasma/serum samples from renal transplant recipients [10] and the use of polyclonal anti-renalase antibodies for characterization of hRenalase1 expression [11,12] may reflect the presence of more than one hRenalase isoform.

The situation could be clarified by producing preparative quantities of renalase isoforms. However, the problem of producing these proteins even as recombinant proteins by means of the commonly used prokaryotic and eukaryotic systems is associated with difficulties in isolation of these scarce transcripts from human tissues/cells.

In order to overcome this problem, in this study we have generated full-length coding sequences of human renalase1 (as control for applicability of the selected strategy) and human renalase2 using human genomic DNA as a template and expressed corresponding hRenalase1 and hRenalase2 in a prokaryotic system. We also compared interaction of both renalases with polyclonal antibodies against hRenalase1. To date, this is the first report on synthesis and purification of hRenalase2.

## 2. Results

**Figure 1:**
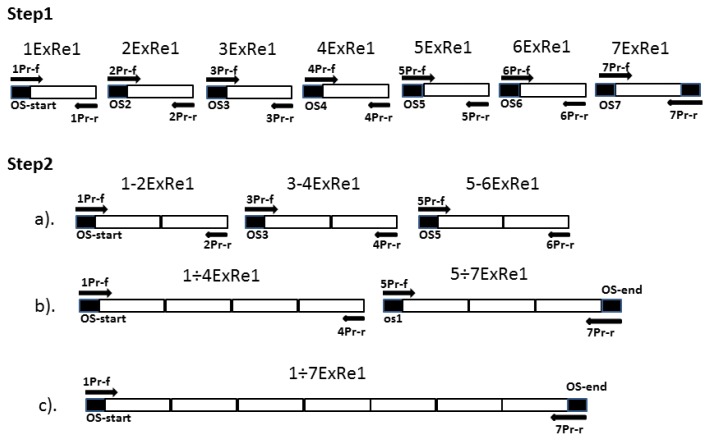
The schematic diagram illustrating the construction of a full-length coding sequence of hRenalases. Step1: Seven individual exons of hRenalase1 (1ExRe1/7ExRe7) with overlapping sequences (OS) were amplified by means of corresponding forward (Pr-f) and reverse (Pr-r) primers; Step2: The amplified exons were sequentially linked together by overlapping PCR. (**a**) 1ExRe1 with 2ExRe1, 3ExRe1 with 4ExRe1, 5ExRe1 with 6ExRe1; (**b**) 1/2ExRe1 with 3/4ExRe1 and exons 5/6ExRe1 with 7ExRe1; (**c**) 1/4ExRe1 with 5/7ExRe1.

Traditional cDNA cloning requires isolation and purification of large amounts of poly-A + mRNA from tissues/cells, which express a gene of interest. Isolation of mRNA of poorly expressible genes represents especially difficult task in the case of human tissues. So we have used another strategy for construction of the coding sequence of hRenalase2, based on assembly of individual exons (Figure 1). Since hRenalase2 differs from hRenalase1 by just one (seventh) exon, it is reasonable to test the employed strategy for hRenalase1, which has been successfully expressed in several independent laboratories [2,5,6,11].

### 2.1. Preparation of Complete Coding Sequence of hRenalase1 and Its Expression in the Prokaryotic System

**Figure 2:**
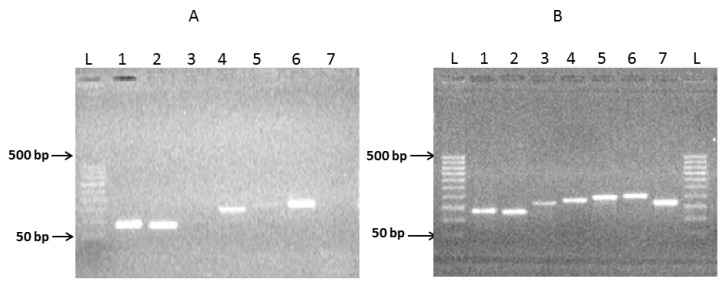
Amplification of individual exons of hRenalase1. (**A**) Agarose gel electrophoresis of PCR products of hRenalase1 exons obtained using the human genomic DNA; (**B**) Step 1 of PCR amplification of hRenalase1 exons using human genomic DNA as a template. The calculated sizes of the amplified exons (corrected for the presence of the OS sequences) were: 1—1ExRe1 (131 bp); 2—2ExRe1 (126 bp); 3—3ExRe1 (166 bp); 4—4ExRe1 (179 bp); 5—5ExRe1 (193 bp); 6—6ExRe1 (197 bp); 7—7ExRe1 (188 bp). L—DNA ladder of 50 bp, 100 bp, 150 bp, 200 bp, 250 bp, 300 bp, 350 bp, 400 bp, 450 bp, 500 bp. Here and in subsequent figures all designations are the same as in Figure 1.

Initially, seven individual exons of hRenalase1 have been amplified by PCR using human genomic DNA as a template (Figure 2A) and rather long forward primers of about 40 nt in length (Table 1). At the 5′-end the forward primers used for the amplification of each exon contained an OS (overlapping sequence) (20 ± 3 nt), which was complementary to the reverse primer of the previous exon, and included a nucleotide sequence of the same length, representing the beginning of the amplified exon. Reverse primers were shorter (about 20 nt) and their sequences were complementary to the nucleotide sequences of the amplified exon starting at its intron-exon border.

Amplification of exons 1, 2, 4, and 6 (1ExRe1, 2ExRe1, 4ExRe1, and 6ExRe1 in Figure 2A) provided a good yield of the amplified products of 131 bp, 126 bp, 179 bp and 197 bp, respectively, with formation of some minor bands (Figure 2A). Amplification of other exons either did not yield visible bands of expected PCR products of 166 bp (3ExRe1) and 188 bp (7ExRe1) or it was very weak (exon 5 (5ExRe1, 193 bp) (Figure 2A) thus suggesting poor amplification. In this case additional re-amplification was performed using the same primers and corresponding PCR products as a template.

**Figure 3:**
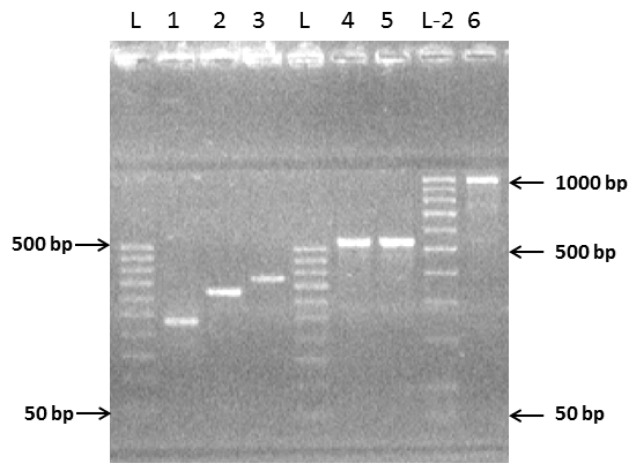
Joining of hRenalase1 exons into the full-length coding sequence. Agarose gel electrophoresis of PCR products of hRenalase1 exons obtained during Step 2. The calculated sizes of the amplified exons (corrected for the presence of the OS sequences) were: 1—1–2ExRe1 (236 bp); 2—3–4ExRe1 (325 bp); 3—5–6ExRe1 (370 bp); 4—1/4ExRe1 (538 bp); 5—5/7ExRe1 (535 bp); 6—1/7ExRe1 (1061 bp). L—DNA ladder (50 bp, 100 bp, 150 bp, 200 bp, 250 bp, 300 bp, 350 bp, 400 bp, 450 bp, 500 bp); L-2—DNA ladder-2 (50 bp, 100 bp, 200 bp, 300 bp, 400 bp, 500 bp, 600 bp, 700 bp, 800 bp, 900 bp, 1000 bp).

During the next step, all amplicons containing seven individual exons (Figure 2B) were used for paired joining of exons as shown in Figure 1 (Step 2). The overlapping PCR-based joining, which included linking of exon1 (1ExRe1 of 131 bp) with exon2 (2ExRe1, 126 bp), exon3 (3ExRe1, 166 bp) with exon4 (4ExRe1, 179 bp), exon5 (5ExRe1, 193 bp) with exon6 (6ExRe1, 197 bp) (Figure 3, tracks 1–3), was carried out using amplicons of both individual exons of each pair and the outmost primers (Table 1). Since electrophoresis in 2% agarose revealed only the PCR-products of expected sizes, it was concluded that the presence of contaminants from the previous step (amplification of individual exons) did not interfere with the exon joining. Consequently, additional purification of amplified individual exons was not necessary.

Subsequent joining of the amplified linked exons 1–2ExRe1 (236, bp) with 3–4ExRe1 (325 bp), and linked exons 5–6ExRe1 (370 bp) with exon 7ExRe1 (188 bp) was carried out by overlapping PCR using the amplified PCR products and corresponding outmost primers (Figure 1 Step 2 b). This resulted in PCR-based synthesis of two nucleotide sequences corresponding in sizes to joint exons 1/4ExRe1 and 5/7ExRe1 (Figure 3, tracks 4,5).

The complete coding sequence of 1/7ExRe1 (1061 bp) (Figure 1, Step 2c) was finally obtained by joining PCR products containing nucleotide sequences of linked exons 1/4ExRe1 (538 bp) with linked exons 5/7ExRe1 (535bp). The overlapping PCR was carried out using amplicons from the previous amplification as template and corresponding outmost primers (Figure 3, track 6).

**Figure 4:**
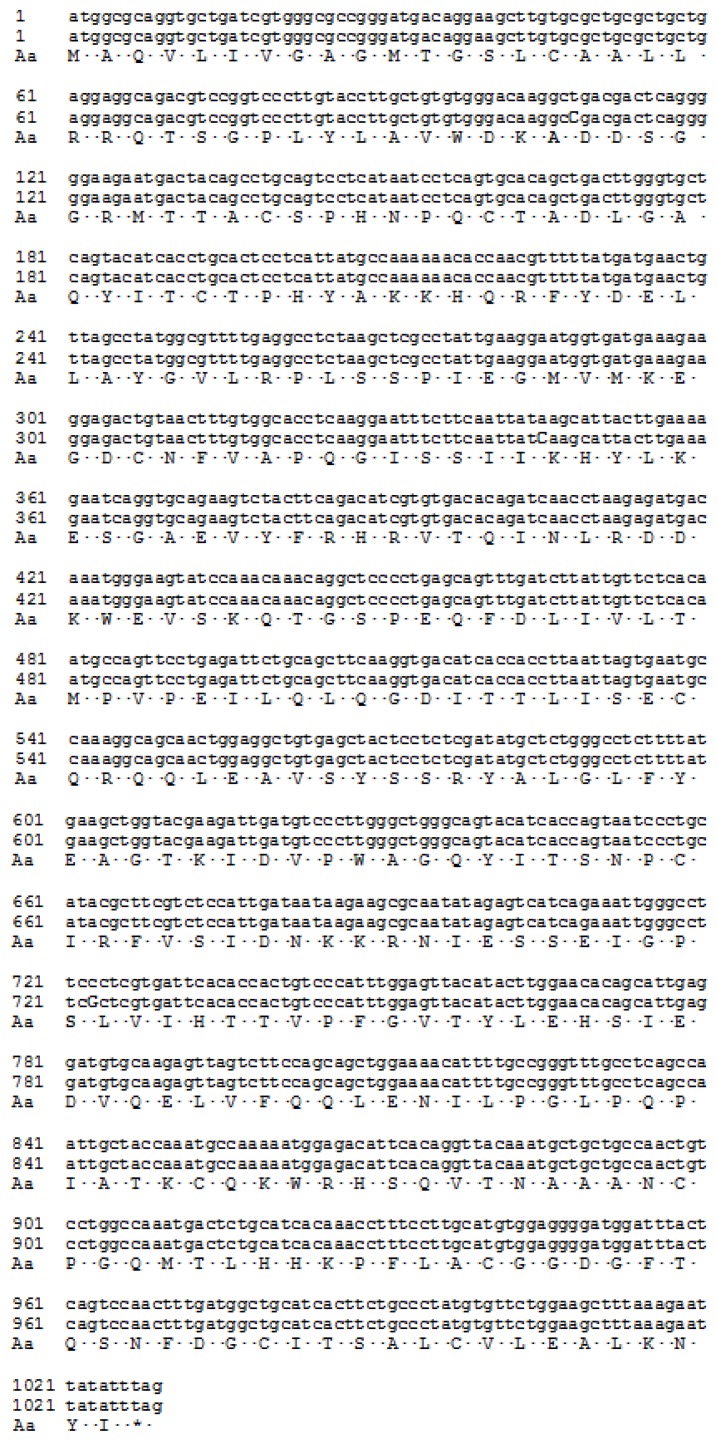
Nucleotide sequences of hRenalase1 ORF available from GenBank (locus NM_001031709.1; upper sequence). hRenalase1 ORF (median sequence) obtained in this study by sequential exon joining, and amino acid sequence of hRenalase1 (lower sequence, a one-letter code). SNPs are shown in bold capital letters.

The resultant complete sequence of hRenalase1 contained the nucleotide sequence of seven exons (1/7) flanking OS-start and OS-end containing restriction sites for restriction endonucleases *Nco*I and *Xho*I, respectively (Figure 3, track 6). It was further amplified, separated by electrophoresis in 2% agarose, excised from the gel, and purified using the Wizard^®^ SV Gel (Promega, Madison, WI, USA) and the PCR Clean-Up System (Promega, Madison, WI, USA). The resultant full-length hRenalase1 coding sequence was inserted in the pGEM^®^-T Easy vector (Promega, Madison, WI, USA), which was then cloned in *Escherichia coli* BL-21 (DE3) cells. Ten cell clones were identified and their plasmids were isolated and subjected to restriction analysis. Sequencing of one plasmid revealed that hRenalase1 coding sequence carried three point mutations at the positions 108, 345, 723, starting from the first AUG codon (Figure 4). These mutations affected the third position of degenerated codons and, consequently, the amino acid sequence of the coded protein remained unchanged: Ala36—GCT to GCC; Ile115—ATA to ATC; Ser241—TCC to TCG. This suggests applicability of the selected strategy for construction of coding sequences of interest.

The full-length hRenalase1 coding sequence was then inserted into the pET-28a(+) vector by *Nco*I and *Xho*I restriction sites and the resultant expression vector pET-hRenI was then transformed into *E. coli* Rosetta (DE3) cells, which were already used for expression of human recombinant hRenalase1 [6].

**Figure 5:**
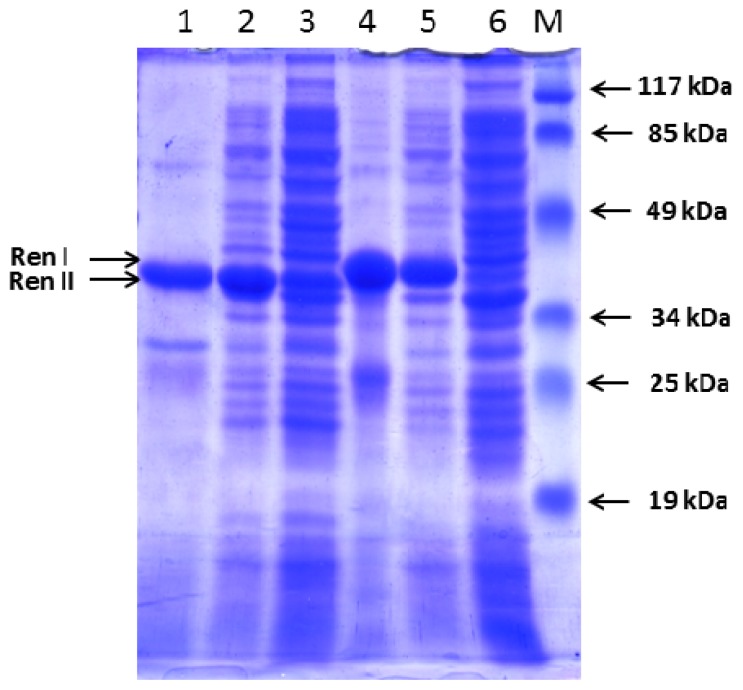
Expression of hRenalase1 and hRenalase2 in *E. coli* cells. Sodium dodecyl sulfate-polyacrylamide gel electrophoresis (SDS-PAGE) analysis of expression of polyHis hRenalase1 (RenI—39 kDa) and hRenalase2 (RenII—36 kDa) in *E. coli* Rosetta (DE3) cells transformed by pET-hRenI and pET-hRenII vectors. Tracks 1 and 4—polyHis hRenalase2 (RenII—36 kDa) and hRenalase1 (RenI—39 kDa), respectively, purified on Ni-Sepharose; Tracks 2 and 5—lysates of cells transformed pET-hRenI и pET-hRenII induced with 1.0 mM IPTG; Tracks 3 and 6—lysates of cells transformed pET-hRenI and pET-hRenII, without IPTG; Track M—molecular mass markers. Mass values are shown on the right.

IPTG induction of *E. coli* Rosetta (DE3) cells transformed with pET-hRenI resulted in production of detectable amounts of a protein with molecular mass of 39 kDa (Figure 5), corresponding to the molecular mass of hRenalase1 (carrying the polyHis tag). In accordance with results by Pandini *et al.* [6] the target protein product (recombinant nRenalase1) was accumulated in Rosetta (DE3) as an insoluble form in inclusion bodies. The purified protein was not subjected to refolding after purification and was directly used for custom-made generation of polyclonal antibodies, which were successfully used for recent mass spectrometry detection of hRenalase1 in human urine [13]. Table 2 summarizes purification steps of both proteins.

### 2.2. Preparation of the Complete Coding Sequence of hRenalase2 and Its Expression in the Prokaryotic System

According to the computational analysis of the renalase gene (http://www.ncbi.nlm.nih.gov/sites/entrez?db=gene&cmd=Retrieve&dopt=full_report&list_uids=55328) [8], the coding sequence of the transcript variant of hRenalase2 differs in the coding sequence of hRenalase1 by the nucleotide sequence of the seventh exon. In addition, hRenalase2 mRNA contains the GAG codon encoding Glu37, while hRenalase1 has structurally related Asp at this position (encoded by GAC). Since this substitution involves exon1 (1ExRe2) we have modified the first reverse and the second forward primers for amplification of exons 1 and 2 (1ExRe2 and 2ExRe2, respectively, Figure 1) for subsequent overlapping PCR. We also synthesized specific primers for amplification of exon7 of hRenalase2 (7ExRe2, Table 1). Other details of synthesis and assembly of the complete coding sequence of hRenalase2 were basically the same as for hRenalase1.

Subsequent sequencing based on insertion of the pGEM^®^-T easy vector (Promega, Madison, WI, USA) carrying hRenalase2 coding sequence into *E. coli* BL-21 (DE3) and all other procedures described for hRenalase1 sequencing (see above) also revealed the presence of the same single nucleotide substitutions; since hRenalase2 mRNA is a bit shorter, their positions have been mapped using the first AUG codon and include the following positions: 108, 345, 723. As in the case of hRenalalase1, these substitutions involved the third position of degenerated codons, and so the amino acid sequence of the coded protein remained unchanged.

The full-length hRenalase2 coding sequence was also inserted into the pET-28a(+) vector by *Nco*I and *Xho*I restriction sites and the resultant expression vector pET-hRenII was then transformed into *E. coli* Rosetta (DE3) cells.

IPTG induction of *E. coli* Rosetta (DE3) cells transformed with pET-hRenII resulted in production of detectable amounts of a protein with molecular mass of 36 kDa (Figure 5), corresponding to the molecular mass of hRenalase2 (carrying the polyHis tag). As in the case of recombinant hRenalase1, synthesized hRenalase2 was expressed in the bacterial host in an insoluble form accumulated in the inclusion bodies.

**Figure 6:**
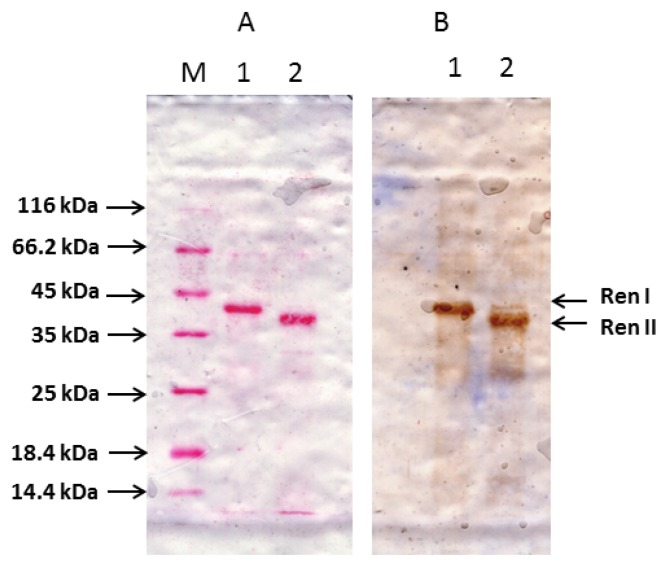
Western blot analysis of hRenalase1 and hRenalase2 using sheep polyclonal antibodies against hRenalase1. (**A**) Staining of the nitrocellulose membrane with Ponceau S after electrotransfer from the SDS-PAGE gel; Track 1—polyHis hRenalase1 (RenI—39 kDa); Track 2—polyHis hRenalase2 (RenII—36 kDa); Track M—molecular mass marker; mass values are shown on the left; (**B**) Western blot analysis with sheep polyclonal antibodies against hRenalase1.

Purified preparations of hRenalase1 and hRenalase2 have been tested for their interaction with sheep polyclonal antibodies produced against hRenalase1. Results of Western blot analysis clearly indicate that both renalases effectively interact with these antibodies (Figure 6). The latter suggests that changes of the blood renalase content in various groups of patients [3,10] and its expression patterns in some cells [11,12] documented by antirenalase1 antibodies [3,10–12] need subsequent reevaluation using antibodies that specifically recognize different renalases (hRenalase1 and hRenalase2).

## 3. Discussion

There is increasing evidence that renalase may be involved in pathogenesis of various pathological conditions [1–5,9,14,15]. Existence of several transcript variants of the renalase gene [2,9] suggests that different isoforms may play different roles in the body and therefore detectable amounts of corresponding proteins are definitely needed to evaluate their role(s) in the body.

The goal of this study was to obtain a coding sequence of hRenalase2, a poorly expressed transcript of the renalase gene. Until this study, only one protein product, known as hRenalase1, has been found and structurally characterized [6,7]. This protein product was obtained in several laboratories [5–7,11], using traditional approaches for cDNA preparation. Originally, Xu *et al*. [5] amplified the renalase fragment of human cDNA library using outmost primers and cloned it into a plasmid vector. This approach requires a cDNA library, but standard cDNA libraries are frequently enriched with the highly expressed genes from tissues/cells used for preparation of such library [16].

Wang *et al*. used total RNA for PCR-based cDNA amplification of the mouse highly expressed analog of human renalase designated as monoamine oxidase C [17]. However, in the case of poorly expressed hRenalase2, the optimal source of tissues/cells for preparation of corresponding cDNA library remains unknown and thus other strategies are needed to obtain its full-length coding sequence. In this study, we have used exons directly from the genomic DNA and joined them into a complete coding sequence.

Earlier, such an approach was used for effective assembly of exons into complete coding sequences directly from genomic DNA [18–20], and the number of assembled exons varied from two [21] to ten [18]. In the case of multi-exon assembly of coding sequence of low copied human polymeric immunoglobulin receptor (PIGR), the authors initially amplified individual exons using primers covering both exon and adjacent intron boundaries, and only after that they did amplify exons with overlapping nucleotide sequences required for a single-stage assembly of all exons. However, it should be noted that this method gives a rather low yield of the target product and a rather high level of side-product contamination [18]. Other authors employed overlapping PCR strategy for paired exon assembly only after purification of amplified exons [19].

We have used this approach for sequential synthesis of individual exons with overlapping sequences directly from genomic DNA and used them without additional purification. This reduced both time and costs of the whole procedure as no kits for PCR fragment purification were needed. According to our data, the presence of contaminants from the previous step (amplification of individual exons) did not interfere with exon joining. The validity of this approach was originally tested using hRenalase1, which had been cloned and expressed in several laboratories. The resultant coding sequence of hRenalase1 assembled by overlapping PCR corresponded well to the nucleotide sequence available from GenBank (Figure 4). Only three single nucleotide substitutions were found at the positions 108, 345, 723 (starting from the first AUG codon). These mutations affected the third position of degenerated codons and therefore did not influence the amino acid sequence of the coded proteins. Three silent synonymous substitutions GCT to GCC, ATA to ATC, and TCC to TCG could reflect specific features of the genomic DNA in the Russian population. Highly accurate coding sequence synthesis may be attributed to the use of Pfu DNA polymerase which, unlike Taq-DNA polymerase, also exhibits 3′→5′ exonuclease (proofreading) activity and is able to correct effectively nucleotide incorporation errors.

Since hRenalase2 differs from hRenalase1 by the seventh exon (Figure 1) and SNP in the first exon leading to Glu37→Asp37 substitution, the following modifications have been made for synthesis of hRenalase2 coding sequence: (i) modification of the first reverse and the second forward primers for amplification of exons 1 and 2 for subsequent overlapping PCR; (ii) use of specific primers for amplification of exon7 of hRenalase2 instead of hRenalase1-specific primers. Resultant full-length coding sequences of both hRenalase1 and hRenalase2 were used for expression of the recombinant proteins in *E. coli* Rosetta (DE3) cells, and hRenalase1 was then used for generation of polyclonal anti-renalase antibodies, which effectively interacted not only with the purified recombinant hRenalase1, but also with hRenalase1 in urine of volunteers [13]. Results of this study indicate that hRenalase2 also interacts with the polyclonal anti-renalase antibodies (Figure 6). This raises the possibility that determination of blood renalase by ELISA in various groups of patients results in detection, not only of hRenalase1, but possibly of hRenalase2. The latter would explain some difficulties in characterization of the role of renalase in pathogenesis of arterial hypertension [2] and diseases associated with impaired kidney functioning [14,15] by interaction of these antibodies with different renalases differentially expressed/secreted under various pathological states. It is possible that some postulated functions of circulating renalase [2,3] may be attributed to the presence of hRenalase2. Using an anti-renalase(1) monoclonal antibody, Wang *et al*. [11,12] demonstrated high abundance of this protein in various kidney cells. Our results raise a possibility that these antibodies detected not only hRenalase1 but also hRenalase2. The latter is especially important in analyses of clinical samples and their correct interpretation. Therefore, the possible clinical relevance not only of hRenalase1, but also hRenalase2 can be demonstrated only by using isoform-specific antibodies, which most likely will be monoclonal antibodies directed against differential isoform-specific epitopes of renalase proteins. It will be also interesting to test in the future, the *in vivo* effects of isoform 2, and to see whether or not it has different properties from isoform 1.

## 4. Experimental Section

### 4.1. Materials

The following reagents were used in this study: Pfu Turbo DNA-polymerase (Stratagene, La Jolla, CA, USA), restriction endonucleases *Nco*I, *Xho*I and molecular mass markers (Fermentas, Vilnus, Lithuania), Ni–Sepharose (GE Healthcare, Stockholm, Sweden), urea (Amresco, Solon, OH, USA). The Wizard^®^ SV Gel and PCR Clean-Up System were from Promega (Madison, WI, USA). The TA cloning vector (pGEM^®^-T easy vector) (Promega, Madison, WI, USA) was purchased from Promega Biotech Co. (Madison, WI, USA). The plasmid pET-28a(+) for protein expression system was obtained from Novagen (Prudhoe, UK). Oligonucleotides for PCR-based synthesis of the coding sequences of hRenalases 1 and 2 (Table 1) were synthesized and purified by polyacrylamide gel electrophoresis by Syntol (Moscow, Russia). A custom polyclonal sheep antibody was raised against human recombinant renalase1 and purified by Pocard Ltd. (Moscow, Russia). A monoclonal anti-rabbit/sheep IgG antibody conjugated with horseradish peroxidase was from IMTEK (Moscow, Russia). The *E. coli* strains Rosetta (DE3) and BL21 (DE3) were obtained from Novagen (Prudhoe, UK). Other chemicals except stated were obtained from Sigma-Aldrich (Moscow, Russia).

### 4.2. Experimental Design: Construction of a Full-Length Renalase Coding Sequence

The general scheme describing construction of full-length coding sequences of hRenalases 1 and 2 consists of two principal steps (Figure 1). Step 1 includes amplification of seven individual exons with overlapping sequences (OS) from the upstream exon at their 3′-ends. The first exon contains the OS-start site, which includes the *Nco*I restriction site. The last exon contains the OS-end site including the *Xho*I restriction site. In step 2, exons are initially paired (see Step 2a,b, Figure 1) and then joined together into entire coding sequences of hRenalase1 and hRenalase2 (Step 2c, Figure 1). The rationale of this strategy is as follows: since all the exon sequences of the renalase gene do exist in the whole human genome, it is possible to optimize PCR conditions for successful amplification of individual exons using primers containing OS-nucleotide sequences at their 3′-ends and to join in the entire coding sequences, applicable for plasmid construction and subsequent protein expression.

### 4.3. Preparation of the Genomic DNA Template

The human genomic DNA was extracted by the conventional method of phenol-chloroform extraction [22,23] from 0.5 mL of whole blood collected from a healthy donor. Briefly, the whole blood sample was mixed with 0.5 mL 20 mM Tris-acetate buffer, pH 7.5, containing 22% sucrose, 20 mM MgCl_2_, 1% Triton X-100, and centrifuged 10 min at 3800 rpm using an Eppendorf Centrifuge 5415R (Hamburg, Germany). The cell sediment was resuspended in the same buffer, diluted twofold with distilled water, and centrifuged as above. The resultant cell sediment was suspended in 0.9 mL of 10 mM Tris-HCl buffer, pH 7.5, containing 400 mM NaCl and 2 mM EDTA. After addition of 0.1 mL 10% SDS and proteinase K (final concentration 1 mg/mL) the resultant mixture was incubated at 58 °C for 3 h under stirring and then sequentially treated with one volume of phenol and then one volume of phenol-chloroform (1:1). After addition of 0.1 volume of 3 M sodium acetate, pH 5.0, and 0.6 volume of isopropanol, DNA was pelleted by centrifugation at 12,000 rpm for 15 min using the same centrifuge (Eppendorf 5415R, Hamburg, Germany) and then washed in cold 70% ethanol. The purified DNA was dissolved in 100 μL of RNase-free water and its concentration was measured by the absorbance at 260 nm using an ultraviolet spectrophotometer Cary 50 (Varian, Palo Alto, CA, USA). The samples were stored at −20 °C until further use.

### 4.4. Exon Amplification and Assembly

The exon amplification and subsequent assembly by overlapping PCR was performed in individual 500 μL tubes (Eppendorf, Hamburg, Germany) using a Tercyc thermocycler (DNA Technology, Moscow, Russia). Each exon amplification was carried out in the reaction mixture (final volume of 20 μL) containing 1× Pfu buffer, 50 ng genomic DNA, 3 μM of each overlapping forward (P-f) and reverse (P-r) primers (see Table 1) and 0.5U Pfu Turbo DNA polymerase (Stratagene, La Jolla, CA, USA). The PCR cycling profile was as follows: pre-denaturation at 95 °C for 5 min, followed by 30 repeated cycles of 20 s at 92 °C, 15 s at 55 °C, 60 s at 72 °C, with a final extension of incubation at 72 °C to 5 min.

Re-amplification was performed in the same reaction mixture (20 μL) and using the same PCR cycling profile as above with only one exception: Genomic DNA was replaced by a PCR-amplificate (2 μL) from the previous PCR step.

### 4.5. Plasmid Construction Cloning and Sequencing

The resultant full-length coding sequence (Figure 1, Step 2c) was purified by electrophoresis in 2% agarose gel using the Wizard SV Gel and PCR Clean-Up System (Promega, Madison, WI, USA) according to manufacturer’s protocols. The purified product was cloned into the plasmid vector pGEM-T using the “Protocol for Ligations Using the pGEM^®^-T and pGEM^®^-T Easy” from Promega (Madison, WI, USA). The correct joining of the coding sequence of the renalase gene was verified by automated DNA sequencing by capillary electrophoresis using a 310 DNA analyzer (Applied Biosystems, Foster City, CA, USA) and the BigDye Terminator v 3.1 Cycle Sequencing Kit (Applied Biosystems, Foster City, CA, USA) as recommended by suppliers. Resultant DNA sequences were compared with known DNA encoding sequences for hRenalase1 and hRenalase2 using the BLAST software (National Center for Biotechnology Information, Bethesda, MD, USA) available at http://www.ncbi.nlm.nih.gov [24].

The coding sequences of hRenalase1 and hRenalase2 were then excised from pGEM-T as a *Nco*I/*Xho*I fragment and inserted between the same sites of pET-28a(+) (Novagen, Prudhoe, UK) containing the T7 promoter, lac-operator and a ribosome binding site. The resulting plasmids, pET-RenI and pET-RenII, directed the synthesis of two fusion proteins, hRenalase1 and hRenalase2, respectively, with a polyHis-tag connected to C-terminus.

### 4.6. Renalase Expression in Prokaryotic Cells and Purification

The constructed plasmids pET-RenI and pET-RenII were transformed into *E. coli* Rosetta (DE3) host cells cultured in the Lysogeny broth (LB) medium containing 50 μg/L kanamycin. The transformed cells were seeded onto LB agar plates containing 50 μg/mL kanamycin. After overnight incubation at 37 °C individual colonies were inoculated into 4 mL of LB-medium containing 50 μg/mL kanamycin and incubated overnight at 37 °C.

The overnight cultures were then inoculated into 200 mL of LB medium containing 50 μg/mL kanamycin in a thermostat shaker (Ecotron, Infors, Lausanne, Switzerland) at 37 °C and 250 rpm. When the cultures reached an OD600 of ~0.6, they were induced with 1.0 mM IPTG (final concentration 1 mM) and incubated for 4 h under the same conditions. Cells were harvested by centrifugation at 5,000× *g* at 4 °C for 20 min using an Avanti J-E centrifuge (Beckman Coulter, Brea, CA, USA) and stored at −20 °C.

The presence of the target product (human recombinant renalase1 or 2) was evaluated in the whole-cell lysates by 15% SDS-PAGE and visualized by Coomassie Brilliant Blue R-250 (Sigma, Moscow, Russia) staining.

The biomass obtained from 2 L of the culture medium (about 30 g) was used for purification of target proteins by affinity chromatography on Ni-Sepharose (10 g) (GE Healthcare, Stockholm, Sweden). Briefly, the biomass was resuspended using overnight shaking (120–160 rpm, Ecotron shaker, Lausanne, Switzerland) in 0.5 L of buffered solution of 8 M urea (Amresco, Solon, OH, USA) containing 0.1 M NaH_2_PO_4_, 10 mM Tris, pH 8.0. The resultant lysate was cleared by two sequential centrifugations for 30 min at 16,000× *g* (Avanti J-E centrifuge, Beckman Coulter, Brea, CA, USA) at room temperature. The cleared supernatant lysate was transferred into a clean flask, mixed with 20 mL of the Ni-Sepharose suspension (GE Healthcare, Stockholm, Sweden) and incubated at room temperature for 2 h under mild shaking (40 rpm). The resultant sorbent suspension was loaded onto a column connected with the BioLogic LP low pressure chromatography system (Bio-Rad, Dreieich, Germany). The sorbent was washed with the same buffer as used during biomass processing, but at pH of 6.3 (1 L) and then at pH of 5.9 (100 mL). Proteins were eluted using the same buffer at pH of 4.5. Preparations of purified recombinant proteins were transferred into 50 mM Tris-HCl (pH 9.0) by stepwise dialysis at 4 °C against 50 mM Tris-HCl buffer, pH 9.0, containing 6, 4, 2, and 1 M urea, and finally against urea-free 50 mM Tris-HCl buffer, pH 9.0.

Concentrations of the purified recombinant proteins were determined spectrophotometrically at 280 nm by using the molar extinction coefficients deduced from the amino acid sequences by the OMIGA 2.0 software (Oxford Molecular Group Inc., Oxford, UK) [25].

### 4.7. Western Blotting

Western blotting was performed using the standard protocol described in [26]. Blotted nitrocellulose membranes were immunostained with the chromogenic substrate 3,3′-diaminobenzidine using an anti-hRenalase1 sheep primary antibody and a monoclonal anti-rabbit/sheep IgG antibody conjugated with horseradish peroxidase following sup

## 5. Conclusions

In this study, we have used PCR-based amplification of individual exons and their joining by overlapping PCR for preparation of full-length coding sequence of humen renalase2 and its subsequent expression in *E. coli* cells. To date, this is the first report on synthesis and purification of hRenalase2. The utility of this approach was initially verified for hRenalase1, which had been earlier expressed in several independent laboratories [2,5,6,11]. Although particular human cells expressing transcription variant 2 of the renalase gene still remain unknown, we do believe that results of our study will attract attention to the renalase mRNA species of about 2.4 kb found by Xu *et al*. [5] in human muscle cells. These are the first candidates of particular human cells preferentially expressing hRenalase2. Identification of the human cells expressing hRenalase2 will help to understand mechanisms responsible for tissue specificity of expression of various transcription variants of the human renalase gene. We do believe that this approach has wider applicability and may be used for construction of coding sequences of various (especially weakly expressible) genes, their transcript variants, *etc*.

## Figures and Tables

**Table 1:** Primers used for overlapping PCR.

Exons	Primers forward (5′-3′)	Primers reverse (5′-3′)
1ExRe1	1Pr-f: cgtcg**ccatgg**cgcaggtgctgatcg	1Pr-r: cctgagtcgtcagccttgtcc
2ExRe1	2Pr-f: ggacaaggctga**C**gactcagggggaagaatgactacagc	2Pr-r: cgttggtgttttttggcataatg
3ExRe1	3Pr-f: cattatgccaaaaaacaccaacgtttttatgatgaactgttagc	3Pr-r: ctgattctttcaagtaatgc
4ExRe1	4Pr-f: gcattacttgaaagaatcaggtgcagaagtctacttc	4Pr-r: aggtggtgatgtcaccttg
5ExRe1	5Pr-f: caaggtgacatcaccaccttaattagtgaatgccaaaggc	5Pr-r: ctatattgcgcttcttattatc
6ExRe1	6Pr-f: gataataagaacggcaatatagagtcatcagaaattgggc	6Pr-r: cctgtgaatgtctccatttttgg
7ExRe1	7Pr-f: ccaaaaatggagacattcacaggttacaaatgctgctgcc	7Pr-r: gaggag**ctcgag**aatataattctttaaagc
1ExRe2		1PrRe2-r: cctgagtcct**C**agccttgtcc
2ExRe2	2PrRe2-f: ggacaaggctga**G**gactcagggggaagaatgactacagc	
7ExRe2	7PrRe2-f: ccaaaaatggagacattcacaggtaccaagtgctggtgtgattc	7PrRe2-r: gaggag**ctcgag**gatgggaaatccaatcgc

Substituted nucleotides in forward and reverse primers used for amplification of exons 1ExRe2 and 2ExRe2 are shown in bold capital letters. The nucleotide sequences corresponding to the *Nco*I and *Xho*I restriction sites are shown in bold and underlined.

**Table 2:** Purification of human polyHis-renalases 1 and 2.

Protein form	Purification step	Volume (mL)	Protein (mg)	A_280_ nm
hRenalase1	Bacterial culture	2000	3500	nd
	Crude extract	500	2750	nd
	Ni-Sepharose affinity chromatography	22.7	57.2	2.65

hRenalase2	Bacterial culture	2000	3350	nd
	Crude extract	500	2350	nd
	Ni-Sepharose affinity chromatography	19.9	33.75	2.12

nd—not determined.
